# Nonobese NAFLD subjects demonstrate lower risk of metabolic syndrome than obese non-NAFLD subjects: A multicenter cross-sectional study

**DOI:** 10.1097/MD.0000000000044187

**Published:** 2025-08-29

**Authors:** Zih-Chien Lu, Kuan-Yu Lai, Hsian-Han Kao, Wen-Yuan Lin, Tsung-Po Chen

**Affiliations:** a Department of Community and Family Medicine, China Medical University Hospital, Taichung, Taiwan; b School of Medicine, China Medical University, Taichung, Taiwan.

**Keywords:** metabolic syndrome, NAFLD, nonobese NAFLD, obesity

## Abstract

Nonalcoholic fatty liver disease (NAFLD) and obesity are risk factors for metabolic syndrome (MetS). Interaction between NAFLD and obesity might deteriorate the probability of MetS. We aim to evaluate the nonobese NAFLD for the risk of MetS compared with obese non-NAFLD. A population-based cross-sectional study was collected in 3 different hospitals in Taiwan from 2015 to 2022. Anthropometric and biochemical measurements were collected after 8-hour fasting. NAFLD was diagnosed through abdominal ultrasonography by trained doctors. Participants were divided into 2 groups by body mass index with a 25 kg/m^2^ cutoff value and NAFLD status. The risk of MetS with the status of NAFLD and obesity was assessed using a multiple logistic regression model. Six hundred ninety eligible participants were involved in the analysis, with 441 nonobese NAFLD and 249 obese non-NAFLD subjects. The mean age was 62.7 ± 16.7 years old. The prevalence of MetS was 35.4% in the nonobese NAFLD group and 47.7% in the obese non-NAFLD group, respectively. The nonobese NAFLD group demonstrates a lower percentage of abnormal waist circumference and blood pressure than the obese non-NAFLD group (*P* < .05). However, the ratio of abnormal triglyceride, high-density lipoprotein cholesterol, and glucose were similar between the 2 groups (*P* > .05). The nonobese NAFLD group demonstrates a lower risk of MetS than the obese non-NAFLD group (odds ratio 0.68, 95% confidence interval: 0.47–0.97). Both NAFLD and obesity increase the risk of MetS. As a risk factor, NAFLD reveals a lower MetS risk than obesity.

## 1. Background

Nonalcoholic fatty liver disease (NAFLD) stands for a condition in which excess fat accumulates in the liver of people who drink little or no alcohol.^[1]^ NAFLD is the most common cause of liver disease in developed countries and affects people of all ages, including children. It is often associated with obesity, type 2 diabetes, high cholesterol, and high triglycerides. The prevalence of NAFLD is increasing globally, with recent estimates indicating that approximately 30% to 32% of adults worldwide are affected, driven by rising rates of obesity and type 2 diabetes.^[2]^ Projections suggest a continued rise, underscoring NAFLD as a major public health challenge.^[3,4]^ The prevalence is even higher in specific populations, such as those with metabolic syndrome (MetS).^[5,6]^ The growing burden of NAFLD is a significant public health concern, as it increases the risk of severe health problems such as liver cirrhosis, liver cancer, and cardiovascular disease.

NAFLD and obesity are closely intertwined risk factors for MetS, sharing common pathophysiological pathways such as insulin resistance, systemic inflammation, and dyslipidemia. These conditions may synergistically exacerbate metabolic dysregulation, increasing the likelihood of MetS. Understanding the interaction between NAFLD and obesity is critical, as their combined effect may amplify the risk of cardiovascular disease and type 2 diabetes, key components of MetS.

Lean or nonobese NAFLD refers to a type of NAFLD in which an individual has an accumulation of fat in their liver but is not overweight or obese.^[7]^ The prevalence of lean NAFLD needs to be better established and can be challenging to quantify accurately. Some studies suggest that lean NAFLD may account for a small percentage of all NAFLD cases, while others report higher rates.^[8–11]^ A meta-analysis of studies conducted in Asia found that lean NAFLD accounted for approximately 15% of all NAFLD cases. In contrast, a similar European analysis found that the percentage of lean NAFLD cases was lower, at around 6%.^[8]^

MetS is a cluster of metabolic risk factors that occur together and increase the risk of developing cardiovascular disease and type 2 diabetes.^[12]^ MetS is a common condition affecting 1 in 4 adults in the United States.^[13]^ It is associated with an increased risk of developing cardiovascular disease, type 2 diabetes, and other health problems, including NAFLD. Maintaining a healthy lifestyle, including a balanced diet, regular physical activity, and a healthy weight, can help prevent or manage MetS.

Both NAFLD and obesity are risk factors for MetS. To address the gap in understanding the distinct metabolic risks posed by nonobese NAFLD, this study evaluates its association with MetS compared to obese non-NAFLD individuals. Emphasizing this comparison is crucial, as nonobese NAFLD represents an underrecognized subgroup with potentially unique risk profiles, necessitating targeted public health interventions to mitigate MetS and its complications.

## 2. Methods

### 2.1. Study population

This study was conducted in the community of Taipei city, Taichung city and Hsinchu city from 2014 to 2021 in Taiwan. Participants with significant alcohol intake (more than 10 g/day for women and more than 20 g/day for men), positive serologic markers for hepatitis B or C virus, history of malignancy, history of autoimmune liver disease, and the use of medications known to cause steatosis, such as amiodarone and corticosteroids, in the past 1 year were excluded from our study. Exclusion criteria were assessed via a standardized questionnaire administered by trained examiners, with participants reporting daily alcohol consumption in grams. Hepatitis B/C serology, malignancy, autoimmune liver disease, and medication history (e.g., amiodarone, corticosteroids) were verified through medical records and laboratory tests. The study was performed in agreement with the Declaration of Helsinki and was approved by the Institutional Review Board of National Taiwan University Hospital (IRB No. 201210012RIC, 201705073RINC) and China Medical University Hospital (IRB No. CMUH110-REC2-064). Informed consent to participate was obtained from all of the participants in the study.

### 2.2. Data collection

A standardized questionnaire was used to gather information on the lifestyle factors and medical history of participants, including the presence of hypertension, type 2 diabetes, dyslipidemia, and thyroid disease. The information was collected by well-trained examiners who asked questions about alcohol consumption, cigarette smoking, and physical exercise. The smoking status were categorized as “nonsmoker,” “current smoker,” and “previous smoker,” respectively. Exercise habits were categorized as “≥ 150 min/week” or “< 150 min/week.” Participants underwent routine physical exams, which included measurements of anthropometric and metabolic data after an overnight fast of 8 hours. The data collection process and detail were described thoroughly in our previous studies.^[14–16]^

### 2.3. Abdominal ultrasonography scanning

Abdominal ultrasonography scans were performed after 8 hours of fasting using a high-resolution B-mode scanner (Hitachi Aloka ProSound α6, Aloka Co., Ltd, Tokyo, Japan or GE Voluson E8, GE Healthcare Austria GmbH & Co OG, Zipf, Austria) equipped with a 3.5 to 5 MHz transducer. The ultrasonography fatty liver index (US-FLI), a semiquantitative score ranging from 0 to 8, was used to assess NAFLD based on features such as liver brightness, hepatorenal echo contrast, deep beam attenuation, and vascular blurring.^[17]^ The detail of US-FLI indicator was described in our previous study.^[14,15]^ A score of 2 or higher was used to diagnose NAFLD, which has been shown to have a high accuracy in detecting steatosis ≥ 10% in histology, with a sensitivity of 90.1% and a specificity of 90%. The scans were performed by 3 experienced physicians in different cities, who were trained to use a consistent scoring system for US-FLI and image acquisition.

### 2.4. Definition of MetS and obesity

MetS was defined using the modified National Cholesterol Education Program Adult Treatment Panel III criteria, adapted for Asian populations by incorporating region-specific waist circumference cutoffs (≥90 cm for men, ≥80 cm for women). This modification accounts for ethnic differences in body composition and visceral fat distribution, improving diagnostic accuracy in Asian cohorts. The criteria were based on the following criteria: elevated fasting plasma glucose levels (≥100 mg/dL) or treatment for diabetes; elevated blood pressure (systolic pressure ≥ 130 mm Hg or diastolic pressure ≥ 85 mm Hg) or treatment with antihypertensive medication; low low-density lipoprotein cholesterol (HDL-C) cholesterol levels (<40 mg/dL in men or < 50 mg/dL in women); elevated triglyceride levels (≥150 mg/dL); and waist circumference (≥90 cm in men or ≥ 80 cm in women).

Obesity was defined as a body mass index of ≥ 25 kg/m^2^, which is the proposed cutoff for Asia. Participants were divided into 2 groups: obese non-NAFLD group and nonobese NAFLD group, based on the presence or absence of obesity and NAFLD.

### 2.5. Statistical analysis

Data was summarized as percentages for categorical variables and as the mean ± standard error for continuous variables. Student *t* test was used to analyze continuous variables and chi-squared test was used to analyze categorical variables. The association between MetS and the 2 groups was evaluated using a logistic regression model, controlling for potential confounding factors such as sex, age, smoking status, drinking habits, and exercise status. The risk of MetS was estimated by odds ratios (OR) and 95% confidence intervals (CIs). Results with a two-tailed *P*-value < .05 were considered significant. The analyses were performed using SAS version 9.4 (SAS Inc., Cary).

## 3. Results

A total of 690 participants, including 441 with nonobese NAFLD and 249 with obese non-NAFLD, participated in the study between 2014 and 2021. Table 1 presents their demographic and anthropometric data. The study included 309 men and 381 women, with a mean age of 62.7 ± 16.7 years. The nonobese NAFLD group had significantly lower mean levels of age, waist circumference, body mass index, and systolic blood pressure compared to the obese non-NAFLD group (*P* < .05). On the other hand, the nonobese NAFLD group had significantly higher mean levels of triglycerides and ALT (*P* < .05). The prevalence of MetS was higher in the obese non-NAFLD group (47.7% vs 35.4%, *P* = .003). There were no differences in total cholesterol, HDL-C, low-density lipoprotein cholesterol, diastolic blood pressure, and fasting plasma glucose between the 2 groups.

**Table 1:** The demographic characteristics of all subjects according to the status of NAFLD.

	Total	Nonobese NAFLD	Obese non-NAFLD	*P*-value
	N = 690	N = 441	N = 249	
Male (n, %)	309 (44.8%)	173 (39.2%)	136 (54.6%)	<.0001
Age (yrs)	62.7 ± 16.7	61.6 ± 16.7	64.7 ± 16.7	.02
Waist (cm)	84.8 ± 8.4	81.2 ± 6.5	90.9 ± 7.8	<.0001
BMI (kg/m^2^)	24.4 ± 2.8	22.8 ± 1.6	27.2 ± 2.0	<.0001
Systolic BP (mm Hg)	130.2 ± 16.8	128.5 ± 16.5	133.1 ± 17.0	.0006
Diastolic BP (mm Hg)	76.6 ± 10.9	76.2 ± 10.3	77.3 ± 11.8	.22
Glu-AC (mg/dL)	102.1 ± 24.2	101.9 ± 25.0	102.6 ± 22.7	.73
TCHO (mg/dL)	182.6 ± 35.9	184.1 ± 36.3	179.7 ± 35.0	.14
TG (mg/dL)	120.2 ± 62.8	124.3 ± 66.1	112.1 ± 54.8	.01
HDL-C (mg/dL)	53.1 ± 12.9	53.2 ± 12.9	52.8 ± 13.0	.73
LDL-C (mg/dL)	111.0 ± 32.3	112.4 ± 33.5	108.3 ± 29.7	.11
ALT (IU/L)	21.0 ± 13.3	22.0 ± 13.1	19.0 ± 13.6	.006
MetS (n, %)	254 (39.5%)	151 (35.4%)	103 (47.7%)	.003

ALT = alanine aminotransferase, BMI = body mass index, Glu-AC = fasting glucose, HDL-C = high-density-lipoprotein cholesterol, LDL-C = low-density-lipoprotein cholesterol, MetS = metabolic syndrome, NAFLD = non-alcoholic fatty liver disease, TCHO = total cholesterol, TG = triglyceride.

Table 2 compares the percentage of each metabolic factor between the nonobese NAFLD and obese non-NAFLD groups. The nonobese NAFLD group had a lower percentage of abnormal waist circumference (36.7% vs 77.1%, *P* < .0001) and abnormal blood pressure (61.3% vs 72.4%, *P* = .004) compared to the obese non-NAFLD group. However, the percentage of abnormal triglycerides, abnormal HDL-C, and abnormal glucose were similar between the 2 groups.

**Table 2:** Percentage of abnormal metabolically factors according to obesity and NAFLD.

	Nonobese NAFLD	Obese non-NAFLD	*P*-value
Abnormal waist circumference	161 (36.7%)	192 (77.1%)	<.0001
Abnormal blood pressure	268 (61.3%)	187 (72.4%)	.004
Abnormal Glucose	181 (41.7%)	102 (46.6%)	.24
Abnormal TG	169 (38.9%)	70 (32.0%)	.09
Abnormal HDL-C	113 (26.1%)	46 (21.0%)	.18

HDL-C = high-density-lipoprotein cholesterol, NAFLD = nonalcoholic fatty liver disease, TG = triglyceride.

To assess the risk of MetS in relation to obesity and NAFLD, a multivariable logistic regression analysis was conducted to account for confounding factors, as shown in Table 3. The results showed that the nonobese NAFLD group had a lower risk of MetS compared to the obese non-NAFLD group (OR = 0.68, 95% CI = 0.47–0.97). Age was associated with an increased risk of MetS (OR = 1.05, 95% CI = 1.04–1.07). However, drinking habits (excluding heavy drinking) were associated with a lower risk of MetS (OR = 0.61, 95% CI = 0.38–0.98). In the multivariable logistic regression models, sex, exercise habits, and smoking habits were not found to be significant independent predictors of MetS.

**Table 3:** The odds ratios of obesity and NAFLD for the risk of metabolic syndrome.

	Model 1	Model 2	Model 3
Group			
Obese non-NAFLD	Ref	Ref	Ref
Nonobese NAFLD	0.60 (0.43–0.84)	0.70 (0.49–1.00)	**0.68 (0.47–0.97**)
Sex (ref = men)		0.70 (0.49–0.99)	0.77 (0.52–1.14)
Age		1.05 (1.04–1.06)	1.05 (1.04–1.07)
Smoking (ref = no)			1.41 (0.69–2.86)
Drinking (ref = no)			0.61 (0.38–0.98)
Exercise (ref = no)			1.41 (0.69–2.87)

Bold values indicate statistical significance at *P* < 0.05. NAFLD = nonalcoholic fatty liver disease, Ref = reference.

## 4. Discussion

This study found that 41.7% of people with NAFLD were not obese, while 20.1% of the general population had nonobese NAFLD. Both obese individuals without NAFLD and nonobese individuals with NAFLD had an increased risk of MetS. Obesity was found to be a higher risk factor for MetS compared to NAFLD.

The classical phenotype of NAFLD patients occurs in obese or overweight. Obesity was considered as one of the important risk factors for NAFLD.^[18]^ NAFLD is commonly associated with central overweight or obesity, but it can also occur in lean individuals.^[19]^ The prevalence of lean and nonobese NAFLD was approximately 5.1% and 12.1%.^[20]^ Besides, the prevalence of lean and nonobese NAFLD in the global NAFLD population was 19.2% and 40.8%, respectively.^[8]^ Our study demonstrated that the prevalence of nonobese NAFLD is 41.7% in NAFLD population and 20.1% in the general population. NAFLD is not uncommon in lean individuals and needs more public attention. The definition of lean varies among studies and can be influenced by race, ethnicity, and metabolic risk factors. This leads to varying prevalence of lean NAFLD between populations and regions.^[8]^

Recently, metabolic dysfunction-associated fatty liver disease was advocated for the positive diagnosis of hepatic steatosis instead of excluding other chronic liver diseases for NAFLD diagnosis.^[21,22]^ Overweight/obesity, the presence of type 2 diabetes mellitus, and evidence of metabolic dysregulation are considered positive criteria for metabolic dysfunction-associated fatty liver disease. However, this new definition might neglect the lean or nonobese population. Semmler et al showed that only 52% to 70% of patients with NAFLD meet these proposed criteria.^[23]^ The lean or nonobese population is a unique group and a special phenotypic correlated with MetS. Compared to the standard group without NAFLD, subjects with lean or nonobese NAFLD have a poorer long-term prognosis, similar to those with overweight or obesity.^[24]^ Lean NAFLD is a prevalent and growing global issue, characterized by metabolic complications.^[10]^ Previous research has also demonstrated a higher risk of MetS and cardiovascular disease among those with lean NAFLD compared to the lean group without NAFLD.^[23]^ These findings underscore the need for targeted risk stratification in nonobese NAFLD patients, who may benefit from regular metabolic screening to detect early signs of MetS. Clinically, lifestyle interventions, including dietary modification and increased physical activity, should be prioritized to mitigate metabolic risks in this subgroup. Early identification of lean NAFLD could prevent progression to severe outcomes, such as cardiovascular disease or advanced liver disease, highlighting the importance of integrating NAFLD screening into routine care for at-risk populations.

Previous studies showed an association between increasing severity of NAFLD and a greater risk of MetS.^[16,25]^ NAFLD is typically considered a “hepatic manifestation” of MetS, meaning that it is a consequence of the metabolic abnormalities associated with MetS. NAFLD is considered a precursor of the MetS. Besides, obesity is also a vital risk factor for development of MetS. Obesity usually correlates and interacts with NAFLD. Our study demonstrated that obesity posed a greater risk for MetS than NAFLD. In contrast to our study, Hu et al showed a higher risk for MetS in the nonobese NAFLD instead of an obese non-NAFLD group.^[26]^ However, there is no significant difference between the 2 groups regarding both males and females. The association between nonobese NAFLD and MetS is complex and needs to be fully understood.

MetS, with its components of abdominal obesity, elevated blood pressure, elevated fasting blood sugar, elevated triglycerides, and low levels of HDL-C cholesterol, increases the risk of developing NAFLD. In some cases, individuals with MetS may have little or no liver fat, but still have an increased risk of liver damage and progression to more severe liver diseases due to the metabolic abnormalities associated with MetS. On the other hand, individuals with lean or nonobese NAFLD may also have features of MetS, such as insulin resistance, high levels of inflammation, and a greater risk of cardiovascular disease. In this sense, lean NAFLD can be seen as a subtype of NAFLD that is associated with MetS.

Our study has several limitations that need to be considered. Firstly, the study’s cross-sectional design does not allow us to establish a causal relationship between NAFLD, obesity, and MetS. Secondly, while liver biopsy is considered the gold standard for diagnosing NAFLD, we used abdominal ultrasonography instead. Despite this, ultrasonography is a valuable tool for detecting NAFLD in population-based studies and correlates well with liver steatosis.^[27]^ Additionally, inter-rater and intra-rater reliability of the US-FLI score were not assessed in this study, though prior research reports high inter-observer agreement (k = 0.81–0.88).^[28]^ Furthermore, the study population was recruited from urban hospitals in Taiwan, potentially introducing selection bias and limiting generalizability to rural or non-Asian populations. Unmeasured confounders, such as dietary patterns or socioeconomic factors, may also influence the findings, warranting caution in interpreting the results.

## 5. Conclusions

In conclusion, lean NAFLD and MetS are closely related, and individuals with 1 condition are at an increased risk of developing the other. Identifying and managing these conditions is essential to prevent developing more severe health problems.

## Author contributions

**Conceptualization:** Wen-Yuan Lin, Tsung-Po Chen.

**Formal analysis:** Kuan-Yu Lai.

**Methodology:** Kuan-Yu Lai, Hsian-Han Kao.

**Supervision:** Wen-Yuan Lin, Tsung-Po Chen.

**Writing – original draft:** Zih-Chien Lu.

**Writing – review & editing:** Tsung-Po Chen.
